# Crossed testicular ectopia: Case report with review of literature

**DOI:** 10.1016/j.ijscr.2020.09.071

**Published:** 2020-09-13

**Authors:** Shakir Saleem Jabali, Ayad Ahmad Mohammed

**Affiliations:** Department of Surgery, College of Medicine, University of Duhok, Kurdistan Region, Iraq

**Keywords:** Ectopic testis, Orchiopexy, Inguinal hernia, Müllerian remnants, Empty scrotum, Genital ridge

## Abstract

**Background:**

Crossed testicular ectopia is a rare form of urogenital anomalies in which both testes are migrated and descend through a single inguinal canal, one or both testes may be ectopic in the abdomen, the inguinal region or descent to the hemi-scrotum with empty contralateral hemi-scrotum.

**Case presentation:**

A 1.5-year-old male patient had an empty left scrotum with no palpable testis in the inguinal region with a palpable testis in the right scrotum with palpable other testis in the right inguinal region. Ultrasound examination showed the right testis in the right scrotum and another testis measuring in the right inguinal region giving suggestion of both testes in the right side. During operation two testes was found in the right inguinal region with two small indirect inguinal sacs. Herniotomy was performed for both sacs. The right testis was placed and fixed in the right hemi-scrotum and the left testis was fixed in the left hemi-scrotum through trans-septal window.

**Conclusion:**

During surgery, the surgeon should adopt a conservative approach to preserve the fertility. Trans-septal orchiopexy or extra-peritoneal transposition orchiopexy is advised. In case of persistent Müllerian duct syndrome, preservation of the Müllerian remnants is recommended to preserve blood supply to the vas deferens and testis. Karyotyping may be required in some cases specially if associated with other congenital anomalies of the genitalia. Patients usually need long term follow up because such patients may have future fertility problems and there is an increased risk of the development of testicular cancer.

## Highlights

• In crossed testicular ectopia both testes are migrated and descend through a single inguinal canal.
• Inguinal hernia is always present in the affected side.
• The diagnosed is usually done during surgery.

## 1 Introduction

Crossed testicular ectopia is a rare form of urogenital anomalies in which both testes are migrated and descend through a single inguinal canal, one or both testes may be ectopic in the abdomen, the inguinal region or descent to the hemi-scrotum with empty contralateral hemi-scrotum. The condition was first reported in 1886 by Von Lenhossek. It is termed also as testicular pseudo-duplication, unilateral double testis, and transverse aberrant testicular mal-descent. The exact incidence is now known, and till now less than 150 cases are reported in literature [[1], [2], [3]].

Inguinal hernia is always present in the affected side. This condition is classified into 3 types based on the associated anomalies: type 1which is the commonest type and account for 50% of cases, in this type there is associated inguinal hernia alone, type 2: which comprises around 30% of the cases and is associated with inguinal hernia and Mullerian duct structures whether rudimentary od persistent, and type 3 which comprises 20% of the cases and is associated with inguinal hernia and other anomalies such as hypospadias, scrotal abnormalities, and pseudohermaphroditism [2].

Most cases are usually diagnosed before the age of 4 years and they present with clinical signs of ipsilateral inguinal hernia with absent both testes, but the final diagnosed is done during surgery and are discovered incidentally. This condition may also be associated with other congenital anomalies of the upper or lower urinary tract [2,4].

In some patients the diagnosis may be made preoperatively using imaging techniques particularly MRI, although this is not done routinely [5].

Sometimes patients have an emergency presentation with obstructed inguinal hernia mandating an emergency surgery [5].

The work of this report case has been reported in line with the SCARE 2018 criteria [6].

## 2 Patient information

A 1.5-year-old male patients presented by his parents to the urologic consultation for checkup complaining from empty left scrotum.

### 2.1 Clinical findings

**Fig. 1:**
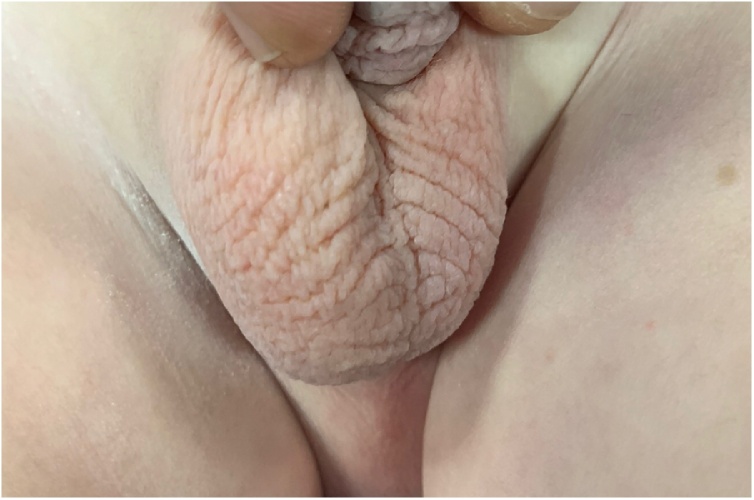
Showing normal right scrotum and empty left scrotum.

The clinical examination showed normal general examination with empty left scrotum, examination of the left inguinal region showed no palpable testis in the inguinal region. In the right side, examination of the right scrotum showed a palpable testis in the right scrotum with palpable anther testis in the right inguinal region Fig. 1.

There was no bulging during crying and the past medical and surgical history was negative.

### 2.2 Diagnostic assessment

**Fig. 2:**
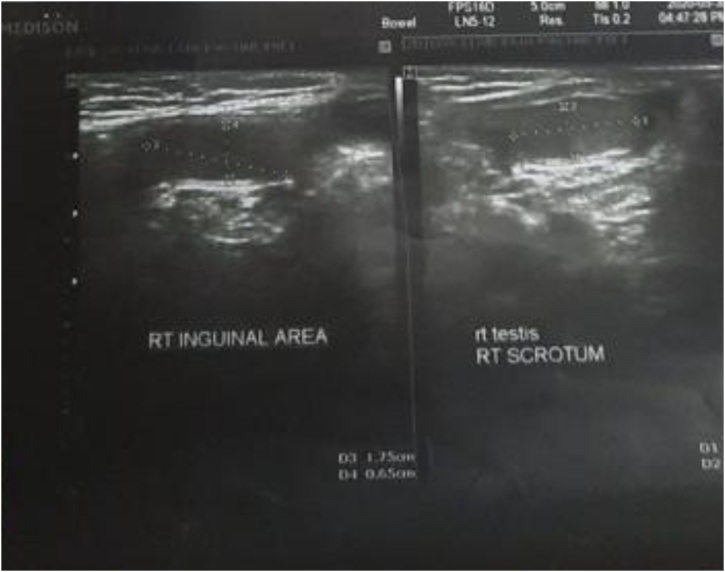
A sonographic picture of the scrotum and the inguinal region showing right testis in the scrotum and left testis in the right inguinal region.

The patients was sent for ultrasound examination which showed the right testis in the right scrotum measuring 16 × 5 mm and other testis measuring 16 × 6 mm in the right inguinal region giving suggestion of both testes in the right side. Fig. 2.

### 2.3 Therapeutic intervention

**Fig. 3:**
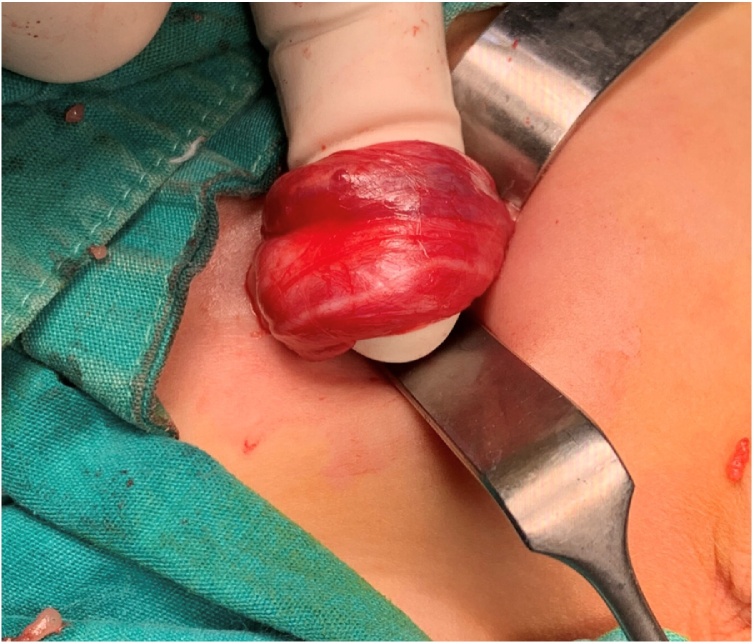
An intraoperative picture showing both spermatic cords in the right inguinal region.

Decision for exploration was done, and during operation two testes were found in the right inguinal region with two small indirect inguinal sacs. Fig. 3.

**Fig. 4:**
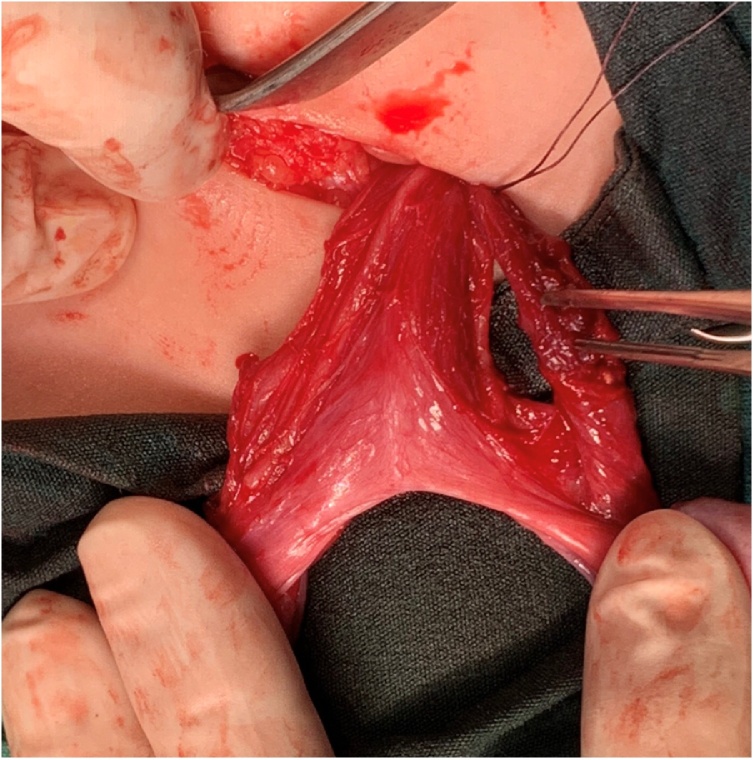
An intraoperative picture showing the dissection of both cords with two separate vas deferenses.

**Fig. 5:**
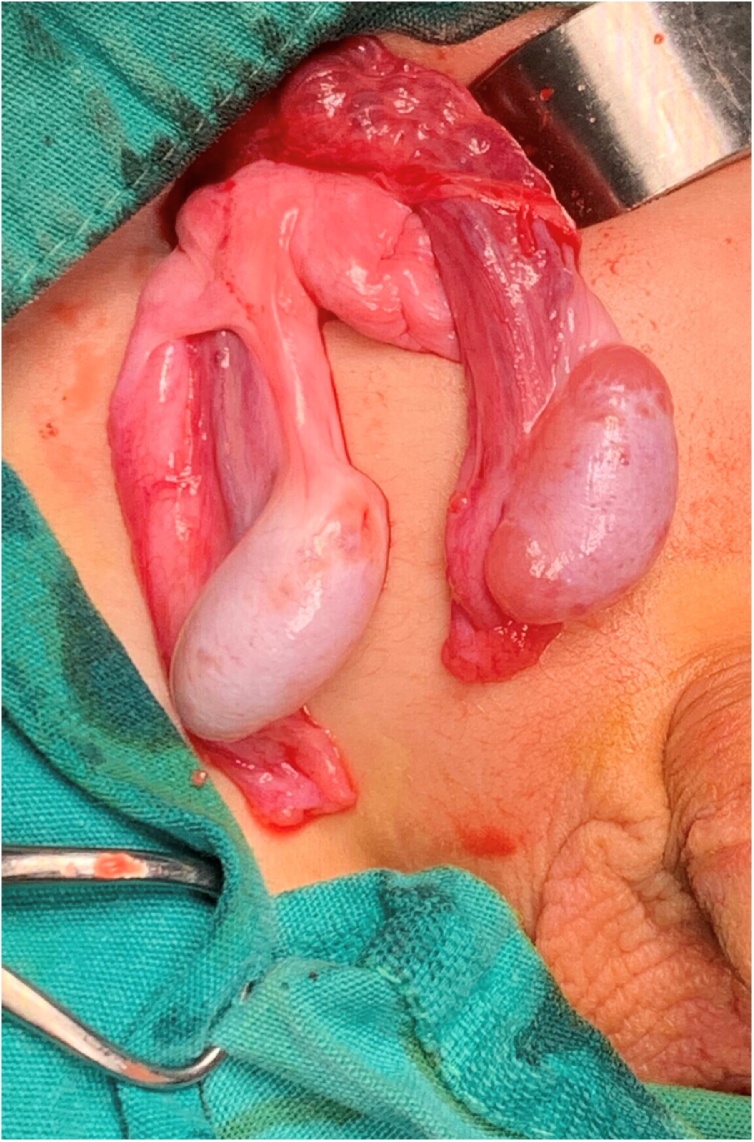
An intraoperative picture showing the two testes and the spermatic cords in the right inguinal region.

**Fig. 6:**
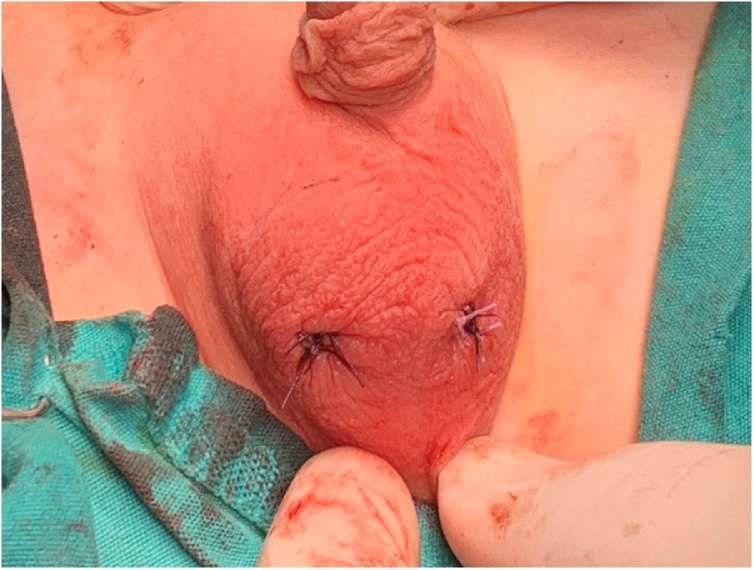
An intraoperative picture showing the scrotum after fixation of both testes in each hemi-scrotum.

Herniotomy was for both sacs. The right testis was placed and fixed in the right hemi-scrotum by a slowly absorbable suture material and the left testis was placed in the left hemi-scrotum through trans-septal window and fixed with slowly absorbable suture material. Fig. 4, Fig. 5, Fig. 6.

### 2.4 Follow-up and outcomes

The patients has uneventful postoperative recovery with no postoperative complications.

## 3 Discussion

The exact etiology of this condition is not well known till now, but some theories are proposing that both testes may arise from the same genital ridge, early adherence and fusion of the developing Wolffian ducts during embryonic development, or one of the testes during its descent causes the second testis to follow it [2].

This condition should be suspected in cases of unilateral inguinal hernia with is non-palpable contralateral testis [7].

There is no any statistical differences in regard to which side is affected more. The preoperative localization of the ectopic testes may be done using ultrasound examination, Ct-scan, MRL, laparoscopy, venography or arteriography in case of very small testis. In our case the diagnosis was made using the ultrasound examination, however during clinical examination both testes were palpated in the right side but the diagnosis was not confirmed [8,9].

During surgery, the surgeon should adopt a conservative approach to preserve the fertility. It is recommended to do orchiopexy by both trans-septal window or extra-peritoneal transposition orchiopexy and Herniotomy. Laparoscopy is helpful too for the diagnosis and the treatment when the testes are not descended to the inguinal canal, it is also helpful to search for other associated anomalies [2,7].

Surgery, in the form of trans-septal orchiopexy or extra-peritoneal transposition orchiopexy is advised. Fusion of the vas deferenses is rare and in such cases trans-septal orchiopexy is recommended. Care must be taken to preserve the blood supply to the vas deferens and testis [3,9,10].

Karyotyping may be required in some cases specially if associated with other congenital anomalies of the genitalia or in case of ambiguous genitalia, in cases of true crossed testicular ectopia the karyotype is always 46XY [8].

Patients usually need long term follow up because such patients may have future fertility problems and there is an increased risk of the development of testicular cancer [2].

## Funding

None.

## Ethical approval

Ethical approval has been exempted by my institution for reporting this case.

## Consent

An informed written consent was taken from the family for reporting the case and the accompanying images.

## Author contribution

Dr Shakir Saleem Jabali and Dr Ayad Ahmad Mohammed contributed to the concept of reporting the case and the patient data recording.

Drafting the work, design, and revision done by Dr Ayad Ahmad Mohammed.

Final approval of the work to be published was done by Dr Ayad Ahmad Mohammed and Dr Shakir Saleem Jabali.

## Registration of research studies

This work is case report and there is no need of registration.

## Guarantor

Dr Ayad Ahmad Mohammed is guarantor for the work.

## Provenance and peer review

Not commissioned, externally peer-reviewed.

## Declaration of Competing Interest

The authors report no declarations of interest.
